# A Deep Generative Model with Multiscale Features Enabled Industrial Internet of Things for Intelligent Fault Diagnosis of Bearings

**DOI:** 10.34133/research.0176

**Published:** 2023-07-07

**Authors:** He-xuan Hu, Yicheng Cai, Qiang Hu, Ye Zhang

**Affiliations:** ^1^ Key Laboratory of Water Big Data Technology of Ministry of Water Resources, Hohai University, Nanjing 211100, P. R. China.; ^2^ College of Computer and Information, Hohai University, Nanjing 211100, P. R. China.

## Abstract

Effective condition monitoring and fault diagnosis of bearings can not only maximize the life of rolling bearings and prevent unexpected shutdowns caused by equipment failures but also eliminate unnecessary costs and waste caused by excessive maintenance. However, the existing deep-learning-based bearing fault diagnosis models have the following defects. First of all, these models have a large demand for fault data. Second, the previous models only consider that single-scale features are generally less effective in diagnosing bearing faults. Therefore, we designed a bearing fault data collection platform based on the Industrial Internet of Things, which is used to collect bearing status data from sensors in real time and feed it back into the diagnostic model. On the basis of this platform, we propose a bearing fault diagnosis model based on deep generative models with multiscale features (DGMMFs) to solve the above problems. The DGMMF model is a multiclassification model, which can directly output the abnormal type of the bearing. Specifically, the DGMMF model uses 4 different variational autoencoder models to augment the bearing data and integrates features of different scales. Compared with single-scale features, these multiscale features contain more information and can perform better. Finally, we conducted a large number of related experiments on the real bearing fault datasets and verified the effectiveness of the DGMMF model using multiple evaluation metrics. The DGMMF model has achieved the highest value under all metrics, among which the value of precision is 0.926, the value of recall is 0.924, the value of accuracy is 0.926, and the value of F1 score is 0.925.

## Introduction

Bearings, as the key components supporting the rotating body in rotating machinery, are widely used in important fields such as transportation, energy, and chemical industry [1–3]. With the improvement of the level of information technology, mechanical equipment is developing toward intelligence and automation, and the requirements for the reliability of rolling bearings are getting higher and higher [4–6]. However, with the accumulation of working hours of mechanical equipment, less than 10% of the bearing failures reach the natural fatigue limit, and most of the rolling bearings fail because of complex and changeable working conditions, which brings immeasurable economic losses or casualties to the society [7–9]. Therefore, effective condition monitoring and fault diagnosis of bearings, timely and reliable assessment of equipment conditions, maintenance, and corrective measures can not only maximize the life of rolling bearings [10–14] but also eliminate unnecessary costs and waste caused by excessive maintenance.

The existing deep-learning-based bearing fault diagnosis models are all implemented on the basis of sufficient training data, and their prediction results are affected by the distribution of abnormal data and normal data [15–19]. In a real factory environment, because of the limitation of the quantity and quality of sensors and collectors, the fault data of various bearings collected by the data collection system will be insufficient. Specifically, when the amount of fault data is small, the prediction effect of the bearing fault diagnosis model based on deep learning will drop obviously. Therefore, how to construct a reliable fault diagnosis model under the condition of small samples is an urgent problem to be solved.

In addition, the existing deep-learning-based bearing fault diagnosis models all use multilayer neural networks to capture the features of fault data [20–24]. These models, which only consider single-scale features, are generally poor in the diagnosis of bearing faults. This is because features of different scales have different advantages. High-level features have strong abstraction ability but are not easy to understand. Low-level features are highly interpretable but have insufficient abstraction ability. Therefore, how to effectively fuse multiscale features is the key to improving the performance of the bearing fault diagnosis model.

In this work, we design a bearing fault data collection platform based on the Industrial Internet of Things (IIoT) [25,26], which is used to collect bearing status data from sensors in real time and feed it back into the diagnostic model. In addition, on the basis of this platform, we propose a bearing fault diagnosis model based on deep generative models with multiscale features (DGMMFs). The DGMMF model augments the bearing data with 4 different variational autoencoder (VAE) models. Specifically, there are 4 kinds of bearing signal data in the dataset, which are normal data, abnormal inner ring, abnormal outer ring, and abnormal rolling. Then, the above 4 kinds of bearing signal data are input into 4 different VAE models for training respectively. Then, from the 4 trained VAE models, the normal data, abnormal inner ring, abnormal outer ring, and abnormal rolling data are respectively sampled and finally inserted into the training set to augment the data samples. The above steps realize the augmentation of normal data and fault data, making the distribution of various samples balanced, thus simplifying the training difficulty of the model.

In addition, the DGMMF model integrates features of different scales. Compared with single-scale features, these multiscale features contain richer information, thereby improving the fault diagnosis performance of the model. Specifically, the DGMMF model uses a multilayer one-dimensional convolutional neural network to extract the high-level features of the bearing data and a single-layer fully connected neural network to extract the low-level features of the bearing data, and then the high-level features and low-level features are fused to obtain multiscale features of bearing data. Finally, the DGMMF model uses the fully connected neural network and multiscale features to predict whether the bearing is faulty and its fault category. We validate the effectiveness of the DGMMF model on a real-world bearing dataset.

The main contributions of this work are as follows:

1. We designed a bearing fault data collection platform based on the IIoT, which is used to collect bearing status data from sensors in real time and feed it back to the bearing fault diagnosis model.
2. We propose a bearing fault diagnosis model DGMMF based on deep generative models with multiscale features. The model can integrate features of different scales and realize the augmentation of normal data and fault data, so that the distribution of various samples is balanced.
3. We conducted a large number of related experiments on the real bearing fault datasets and verified the effectiveness of the DGMMF model using multiple evaluation metrics.

The rest of the content is organized as follows. Related Work introduces the research progress of the bearing fault diagnosis model. Method introduces the bearing fault data collection platform and DGMMF model proposed in this work. Results and Discussion describes the experimental results of the DGMMF model on real bearing datasets. Conclusion summarizes the content of this work and discusses the direction of future improvement.

## Related Work

In this section, we first introduce related work on traditional abnormal detection algorithms. Then, we will introduce the latest research progress of the bearing fault diagnosis model.

### Abnormal detection

Tang et al. [27] proposed a deep-neural-network-based fault diagnosis model for rotating devices. They believe that shallow neural networks are flawed in performance and cannot meet the actual needs of smart devices. Therefore, they utilized more powerful convolutional neural networks, which are good at capturing nonlinear relationships between features. They verified the effectiveness of the model in the experiment.

Ranjith et al. [28] used the density-based spatial clustering of applications with noise (DBSCAN) algorithm to monitor traffic videos to detect abnormal driving and suspicious behavior. The DBSCAN [29] is a density-based clustering algorithm. DBSCAN can be used not only for clustering problems but also for abnormal detection problems. The basic idea is to find low-density abnormal samples in the sample space. The operation of DBSCAN can be decomposed into 4 parts. The first step is to select any data point from the datasets. In the second step, the selected data point is used as the core point, and all data points that can directly reach the core point are found using hyperparameters to form a cluster. The third step is to reselect if the selected data point is an edge point. The fourth step is to repeat the process of steps 2 and 3 until all points are processed.

Yan et al. [30] used autoencoders to detect abnormal timing in video streams. The autoencoder is essentially used for representation learning tasks, that is, to compress the original data points into a low-dimensional vector and then restore the low-dimensional vector to the original data through the decoding operation [31]. The idea of using an autoencoder to handle abnormal detection tasks is as follows. Autoencoder trained on the basis of normal data can reconstruct and restore normal samples, but it cannot restore data points that are different from the normal distribution. Therefore, if a new sample is encoding and, after decoding, its error exceeds the error range of normal data after encoding and decoding, it is regarded as abnormal data.

Zhang et al. [32] used one-class support vector machine (SVM) to monitor intrusion behavior and abnormal traffic in the network. The algorithm idea of one-class SVM [33] is very simple. It is to find a hyperplane to circle the positive examples in the sample. The prediction is to use this hyperplane to make decisions. The samples in the circle are considered positive samples, and the samples outside the circle are negative samples. The idea of applying one-class SVM to abnormal detection tasks is as follows. We regard all abnormal samples as negative samples and all normal samples as positive samples. Then, through positive and negative samples, it is learned that it belongs to an unsupervised learning hyperplane to make a decision, and all samples that are not in the hyperplane are predicted as abnormal data.

### Bearing fault diagnosis

Yang et al. [34] believed that the existing bearing fault diagnosis model based on deep learning directly analyzes the vibration information of the bearing, and such an operation lacks interpretability to a certain extent. Therefore, to increase the interpretability of the bearing fault diagnosis model, they combined convolutional neural network, recurrent neural network, and attention mechanism to realize automatic diagnosis of bearing signals. The reason why the model can be interpreted is that they use the attention mechanism to visualize the weight distribution of the input data, thereby increasing the interpretability.

Li et al. [35] believed that the existing bearing fault diagnosis model based on deep learning did not take into account the difference in distribution between training data and test data. This difference presents a domain shift problem, which leads to severe degradation in the diagnostic performance of the model. Therefore, for this problem, they proposed a domain adaptation method for bearing fault diagnosis. The main structure of the method is a convolutional neural network, and, on this basis, the representation of the source domain is adapted to the target domain by minimizing the average difference. The effectiveness of the domain adaptation method is verified by experiments.

Zhu et al. [36] believed that the fault features extracted by the existing bearing fault diagnosis model were not very effective, which led to the low performance of fault diagnosis. Aiming at this problem, they proposed a model based on feature extraction and fusion to diagnose bearing faults. The idea of the model is as follows. First, the frequency domain features of the bearing signal are transformed into a frequency domain matrix. Then, the features of the matrix are extracted by singular value decomposition and finally input into the SVM to obtain the predicted value.

Patel and Upadhyay [37] proposed a feature ranking and selection model based on Euclidean distance for bearing fault diagnosis. The model can select valuable features from the original feature set and can achieve high-precision fault diagnosis with a small number of features and calculation time. Experimental results on 2 real-world datasets demonstrate the superiority of the model, saving a lot of time for feature selection.

However, some previous research work defined bearing fault diagnosis as a binary classification problem, and it was impossible to infer the faulty bearing category, such as normal status, rolling fault, inner ring fault, and outer ring fault. In this work, we define bearing fault diagnosis as a multiclassification problem, that is, to infer the fault category of the bearing. We can compare various types of bearing faults together and can judge the fault location of the bearing at one time. This saves manpower and material resources to a great extent.

## Method

In this section, we first introduce the architecture of the IIoT-based bearing fault data collection and diagnosis platform. How the VAE model generates fault data and the implementation details of the multiscale features of the DGMMF model is then introduced.

### An IIoT-based bearing fault data collection and diagnosis platform

**Fig. 1.**
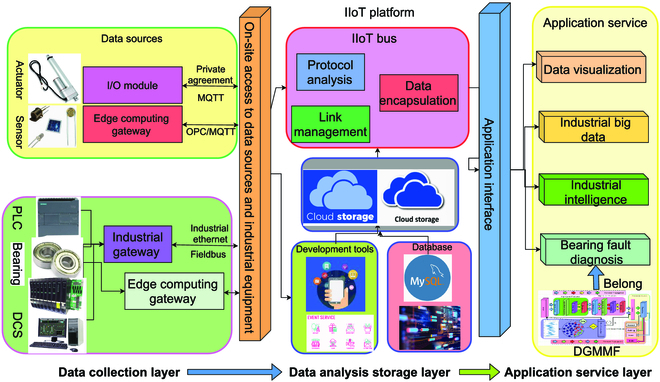
The architecture of the IIoT-based bearing fault data collection and diagnosis platform.

We use the IIoT technology to build an IIoT data collection and diagnosis platform for bearing fault. Figure 1 shows the architecture of the IIoT data platform. From left to right are the data collection layer, data analysis storage layer, and application service layer of the platform. The platform first uses the data collection layer to collect status data from sensors, actuators, and other field devices, including the collection of bearing running data. The role of the data analysis storage layer is to parse the collected data into a human-understandable format and store it in the database. The role of the application service layer is to develop various valuable applications for the status data stored in the database, including the diagnosis of bearing faults.

### Augmenting fault data with deep generative model

In a real factory environment, because of the limitation of the quantity and quality of sensors and collectors, the fault data of various bearings collected by the data collection system will be insufficient. Specifically, when the amount of fault data of various types of bearings is small, the prediction effect of the bearing fault diagnosis model based on deep learning will drop obviously. Hence, we use VAE model to augment the bearing signal data. This is actually a preprocessing operation on the dataset, which can be used to improve the performance of the model. The VAE model needs to augment the data with the signals of the identified bearing fault categories. The augmented data are combined with the original data to train the subsequent DGMMF model. In the experimental part of this work, the public bearing dataset is used. This dataset has known fault categories in advance, so VAE can be used to augment the data samples and then used for training the DGMMF model. In the real factory environment, the factory needs to manually collect the bearing fault category data determined in the machinery and then use the VAE model to augment the collected data to train the DGMMF model. Then, the factory uses the trained DGMMF model to carry out long-term intelligent fault monitoring and diagnosis for the mechanical bearings.

**Fig. 2.**
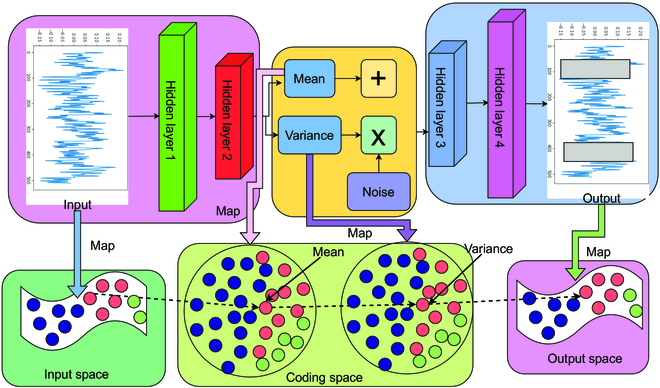
The frame diagram of the VAE model to augment fault data.

In this work, the DGMMF model is augmented with data from 4 different VAE models. Figure 2 is the frame diagram of the VAE model to augment fault data. First, the workflow of VAE is shown in Eqs. 1 to 4.

$$H_{1}=HL_{1}\left(X\right)$$ (1)

$$M,V=HL_{2}\left(H_{1}\right)$$ (2)

$$H_{2}=HL_{3}\left(M,V\right)$$ (3)

$$\hat{X}=HL_{4}\left(H_{2}\right)$$ (4)

Among them, *HL*_1_, *HL*_2_, *HL*_3_, and *HL*_4_ are hidden layers, respectively. *X* represents the time series signal of the bearing. *M* and *V* represent the mean and variance, respectively. A trained VAE model can be learned by minimizing the original data and reconstructing the data. Then, the VAE model is sampled to generate bearing data through Eqs. 5 and 6 and then added to the training set to augment the data samples.

$$\mathrm{Gen}=\mathrm{VAE}\left(S\right)$$ (5)

Input=Gen+Data (6)

Among them, *Data* is the original data, and *Gen* is the generated data. *Input* represents the training set that is finally used to train the model. There are 4 kinds of bearing signal data in the dataset, which are normal data, abnormal inner ring, abnormal outer ring, and abnormal rolling. Then, the above 4 kinds of bearing signal data are input into 4 different VAE models for training. The above steps realize the augmentation of normal data and fault data, making the distribution of various samples balanced, thus simplifying the training difficulty of the model.

### A DGMMF-based bearing fault diagnosis model

**Fig. 3.**
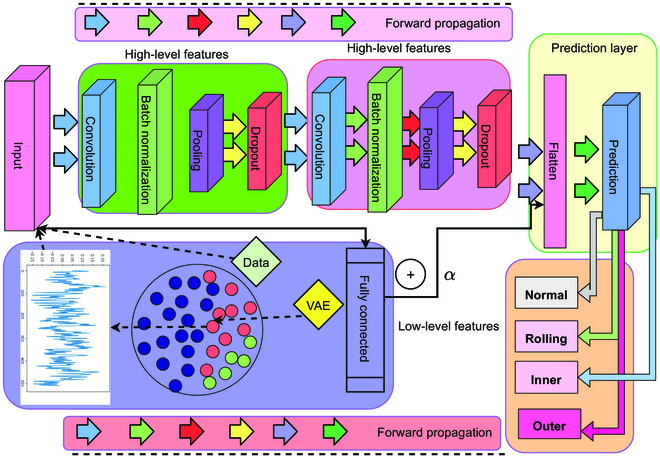
The frame diagram of the DGMMF model.

Relying on the IIoT data collection platform, we propose a bearing fault diagnosis model named DGMMF. Figure 3 is the frame diagram of the DGMMF model. Previously diagnostic models that only consider single-scale features are generally poor in the diagnosis of bearing faults. This is because features of different scales have different advantages, and the abstraction ability of high-level features is strong, but it is not easy to understand. Low-level features have strong interpretability but insufficient abstraction ability. Therefore, the DGMMF model integrates features of different scales. Compared with single-scale features, these multiscale features contain richer information, thereby improving the fault diagnosis performance of the model. Specifically, as shown in Eq. 7, the DGMMF model uses a multilayer one-dimensional convolutional neural network to extract high-level features of bearing data.

$$\mathrm{HF}=\mathrm{Con}1D\left(X^{\prime }\right)$$ (7)

$$\mathrm{Con}1D=\left[C,B,P,D\right]$$ (8)

Among them, *HF* represents the high-level feature, and *X* ^′^ represents the time series signal of the bearing that needs to be diagnosed. *Con*1*D* represents one-dimensional convolution operation, generally using multiple one-dimensional convolutions to extract features. The composition of one-dimensional convolution operation is shown in Eq. 8. Among them, *C*, *B*, *P*, and *D* are respectively represented as convolution operation, batch normalization, pooling operation, and dropout operation. The DGMMF model then utilizes Eq. 9 to extract low-level features.

$$\mathrm{LF}=\mathrm{FC}\left(X^{\prime }\right)$$ (9)

where *LF* represents the low-level features of the bearing and *FC* represents the fully connected network. Then, the DGMMF model uses Eqs. 10 and 11 to fuse low-level features and high-level features to form multiscale features *MSF*.

$$\mathrm{FHF}=\text{Flatten}\left(\mathrm{HF}\right)$$ (10)

MSF=FHF+αLF (11)

where *FHF* is the straightened high-level feature and *Flatten* represents the straightening operation. Finally, the DGMMF model inputs *MSF* into the predictor shown in Eq. 12 and outputs the fault category *Y* of the bearing.

$$Y=\text{Predictor}\left(\mathrm{MSF}\right)$$ (12)

where *Predictor* generally consists of a fully connected network. The value of *Y* is one of normal, rolling, inner ring, and outer ring. The pseudocode of the DGMMF model is shown in Algorithm 1.

**Algorithm 1**. DGMMF.

**Input**: Bearing’s time series signal, *X*

**Output**: Predicted category of the bearing’s time series signal, *Y*

1: Augmenting fault data with deep generative model

2: $H_{1}=HL_{1}\left(X\right)$

3: $M,V=HL_{2}\left(H_{1}\right)$

4: $H_{2}=HL_{3}\left(M,V\right)$

5: $\hat{X}=HL_{4}\left(H_{2}\right)$

6: $\mathrm{Gen}=\mathrm{VAE}\left(S\right)$

7: Input=Gen+Data

8: A DGMMF-based bearing fault diagnosis model

9: $\mathrm{HF}=\mathrm{Con}1D\left(X^{\prime }\right)$

10: $\mathrm{Con}1D=\left[C,B,P,D\right]$

11: $\mathrm{LF}=\mathrm{FC}\left(X^{\prime }\right)$

12: $\mathrm{FHF}=\text{Flatten}\left(\mathrm{HF}\right)$

13: MSF=FHF+αLF

14: $Y=\text{Predictor}\left(\mathrm{MSF}\right)$

15: **Return** *Y*

## Results and Discussion

In this section, we first introduce the bearing fault datasets used, followed by the relevant setup of the experiments. Then, we introduce the influence of the weight parameter *α* in Eq. 11 on the DGMMF model, the influence of the weight decay of the optimizer, and learning rate on the DGMMF model. Finally, we will introduce the comparison of experimental results between the DGMMF model and other models.

### Datasets

**Fig. 4.**
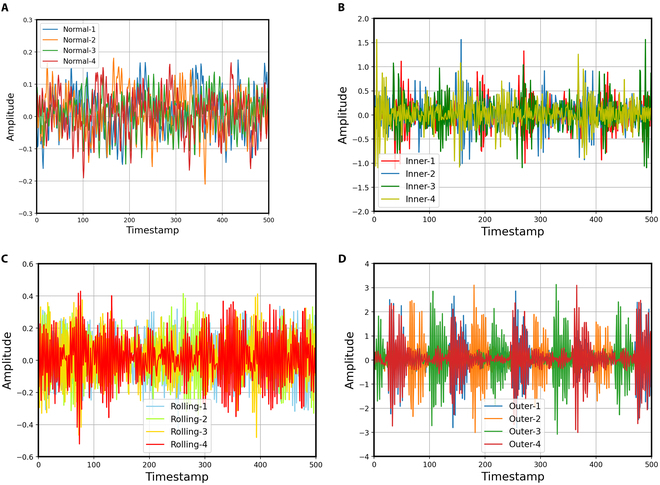
The data distribution of normal and fault data. (A) Normal data, (B) inner ring fault data, (C) rolling fault data, and (D) outer ring fault data.

We use a publicly available bearing failure dataset for this experiment. The diameters of the bearings are 0.007, 0.014, and 0.021 mm, respectively. The status of each bearing is normal status, rolling fault, inner ring fault, and outer ring fault, respectively. Examples of each status are shown in Fig. 4. Figure 4A to D respectively shows 4 normal bearing signals, 4 signals of rolling fault, 4 signals of inner ring fault, and 4 signals of outer ring fault.

### Experimental setup

We slide the window to intercept and sample the time series data. Seventy percent of the data are used as training samples, and 30% of the data are used as test samples. To numerically display the diagnostic ability of the model, we use evaluation metrics such as precision, recall, accuracy, and F1 score to measure the performance of various models.

### The influence of weight parameter *α* on DGMMF model

**Fig. 5.**
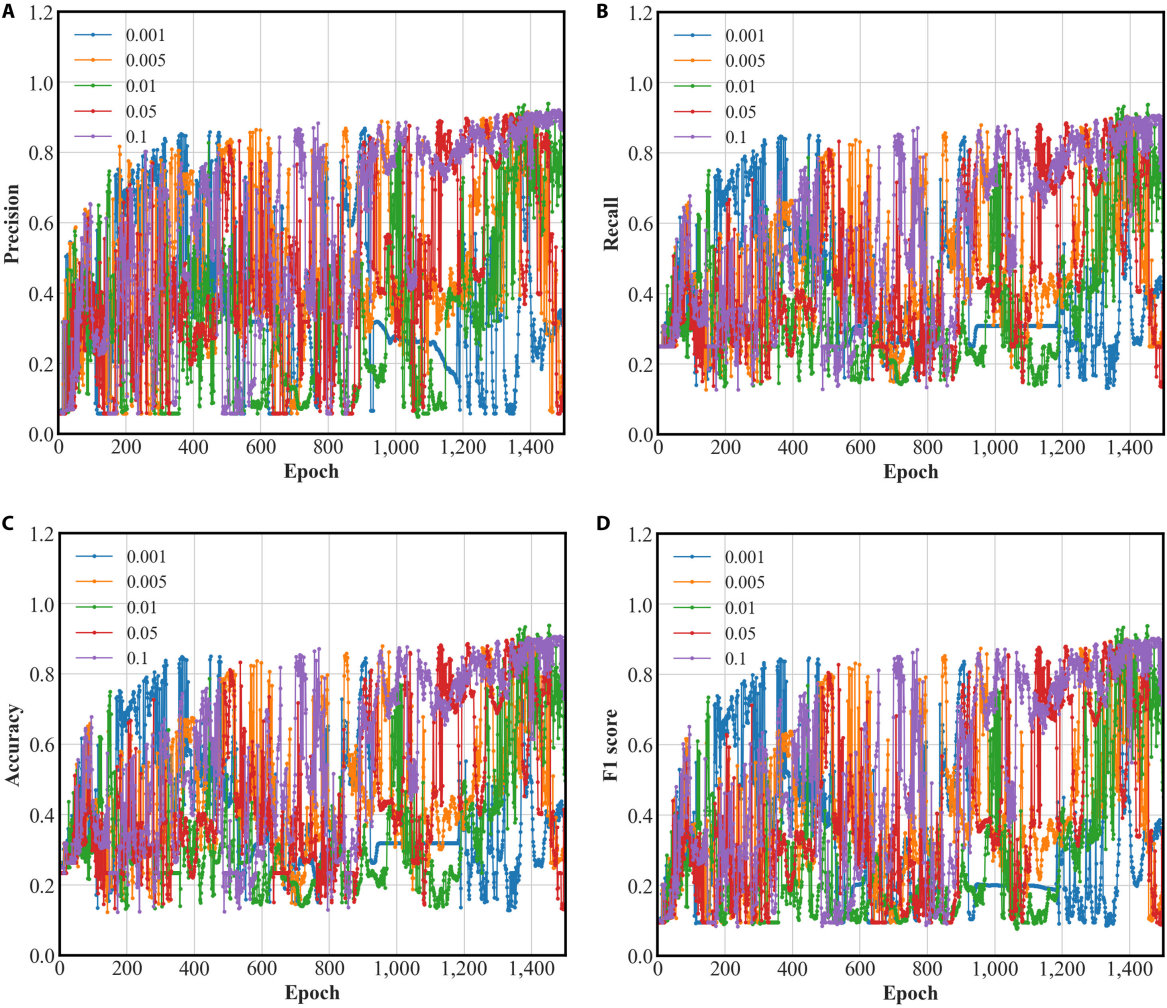
The influence of weight parameter *α* on DGMMF model. (A) Precision, (B) recall, (C) accuracy, and (D) F1 score.

The weight parameter *α* controls the proportion of low-level features in multiscale features. The larger the value of the weight parameter *α*, the higher the proportion of low-level features in multiscale features. The smaller the value of the weight parameter *α*, the lower the proportion of low-level features in multiscale features will be. Therefore, it can affect the amount of information contained in the multiscale features, thereby affecting the diagnostic ability of the subsequent DGMMF model. Therefore, we run the following experiments to compare the diagnostic ability of the DGMMF model under different weight parameters *α*. We manually adjust the size of the weight parameter *α* to obtain different evaluation metrics. The variation range of *α* is [0.001, 0.005, 0.01, 0.05, 0.1]. The experimental results are shown in Fig. 5.

The abscissa in Fig. 5 is the number of iterations of the DGMMF model, and the ordinate is the score of the DGMMF model under different evaluation metrics. The DGMMF model has different diagnostic capabilities under different weight parameters *α*. The above experimental results show that in most cases, the low-level features in the multiscale features are helpful to the diagnostic ability of the DGMMF model. The weight of low-level features in multiscale features needs to be carefully designed. Otherwise, inappropriate weight values will decrease the diagnostic ability of the DGMMF model.

### The influence of weight decay on the DGMMF model

**Fig. 6.**
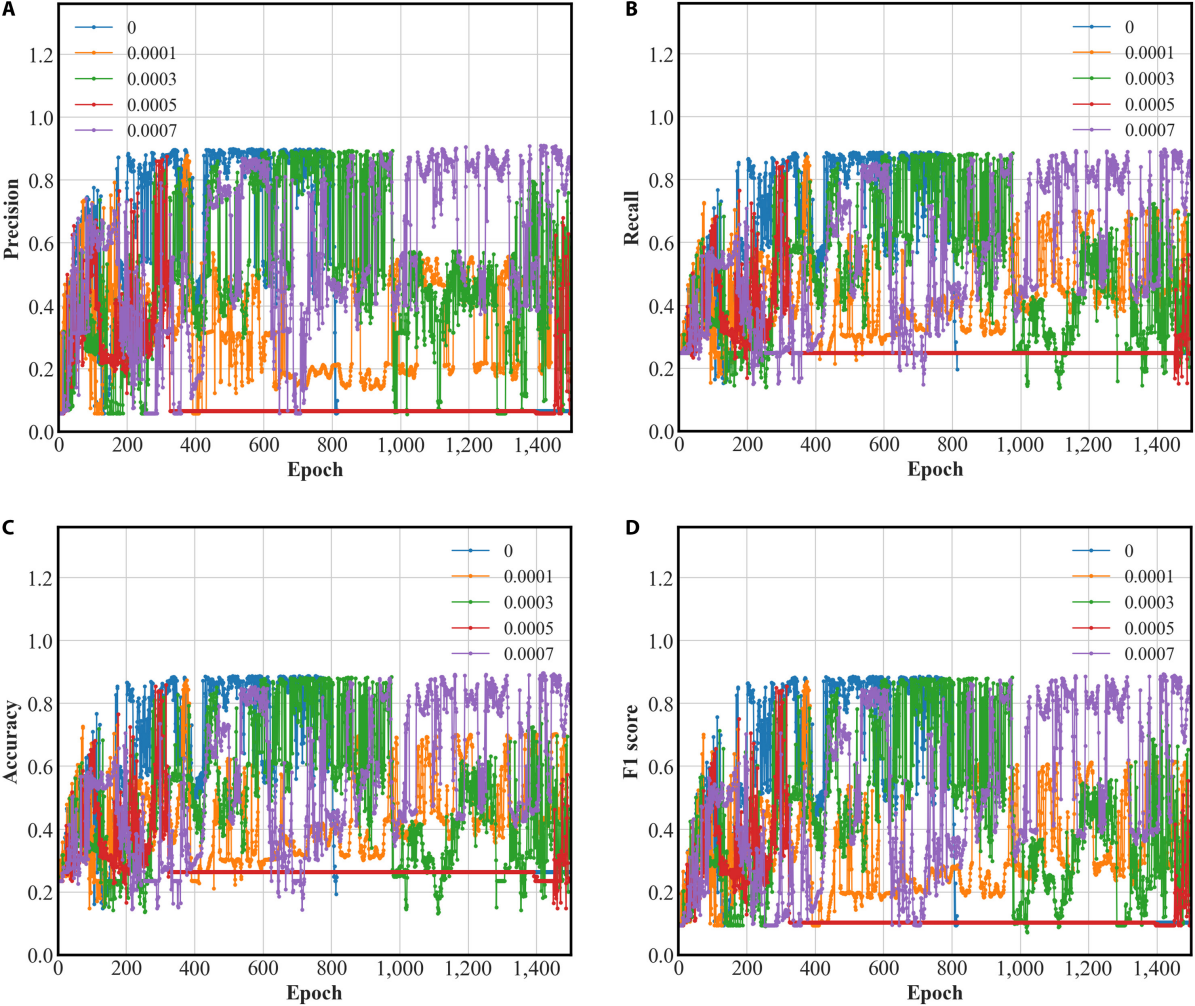
The influence of weight decay on the DGMMF model. (A) Precision, (B) recall, (C) accuracy, and (D) F1 score.

The purpose of weight decay in the optimizer is to prevent overfitting of the DGMMF model. In the loss function, weight decay is a coefficient placed in front of the regular term. The regular term generally indicates the complexity of the model, so the role of weight decay is to adjust the influence of model complexity on the loss function. Therefore, we run the following experiments to compare the diagnostic ability of the DGMMF model under different weight decay. We manually adjust the size of weight decay to obtain different evaluation metrics. The range of weight decay is [0, 0.0001, 0.0003, 0.0005, 0.0007]. The experimental results are shown in Fig. 6.

The abscissa of Fig. 6 is the number of iterations of the DGMMF model, and the ordinate is the score of the DGMMF model under different evaluation metrics. The diagnostic performance of the DGMMF model is different under different weight decay values. We can clearly find that when the value of weight decay is 0.0007, the experimental results of the DGMMF model under the 4 evaluation metrics of precision, recall, accuracy, and F1 score are the best. It can be found that the DGMMF model with a weight decay value of 0.0007 is better than the DGMMF model with a weight decay value of 0, which shows that the weight decay can improve the diagnostic performance of the DGMMF model.

The above experimental results show that an appropriate weight decay value can adjust the influence of the complexity of the DGMMF model on the loss function, thereby avoiding the occurrence of overfitting. Therefore, we need to use the validation set to find the appropriate weight decay value, so as to further improve the diagnostic ability of the DGMMF model. We change the value of weight decay in the interval [0, 0.0001, 0.0003, 0.0005, 0.0007]. We choose the optimal weight decay value based on its performance on the validation set.

### The influence of learning rate on the DGMMF model

We run the following experiments to observe the effect of learning rate on the DGMMF model. The specific settings are as follows, the change interval of the learning rate is [0.001, 0.01, 0.05, 0.1, 0.5]. The evaluation metrics used have mentioned above.

**Fig. 7.**
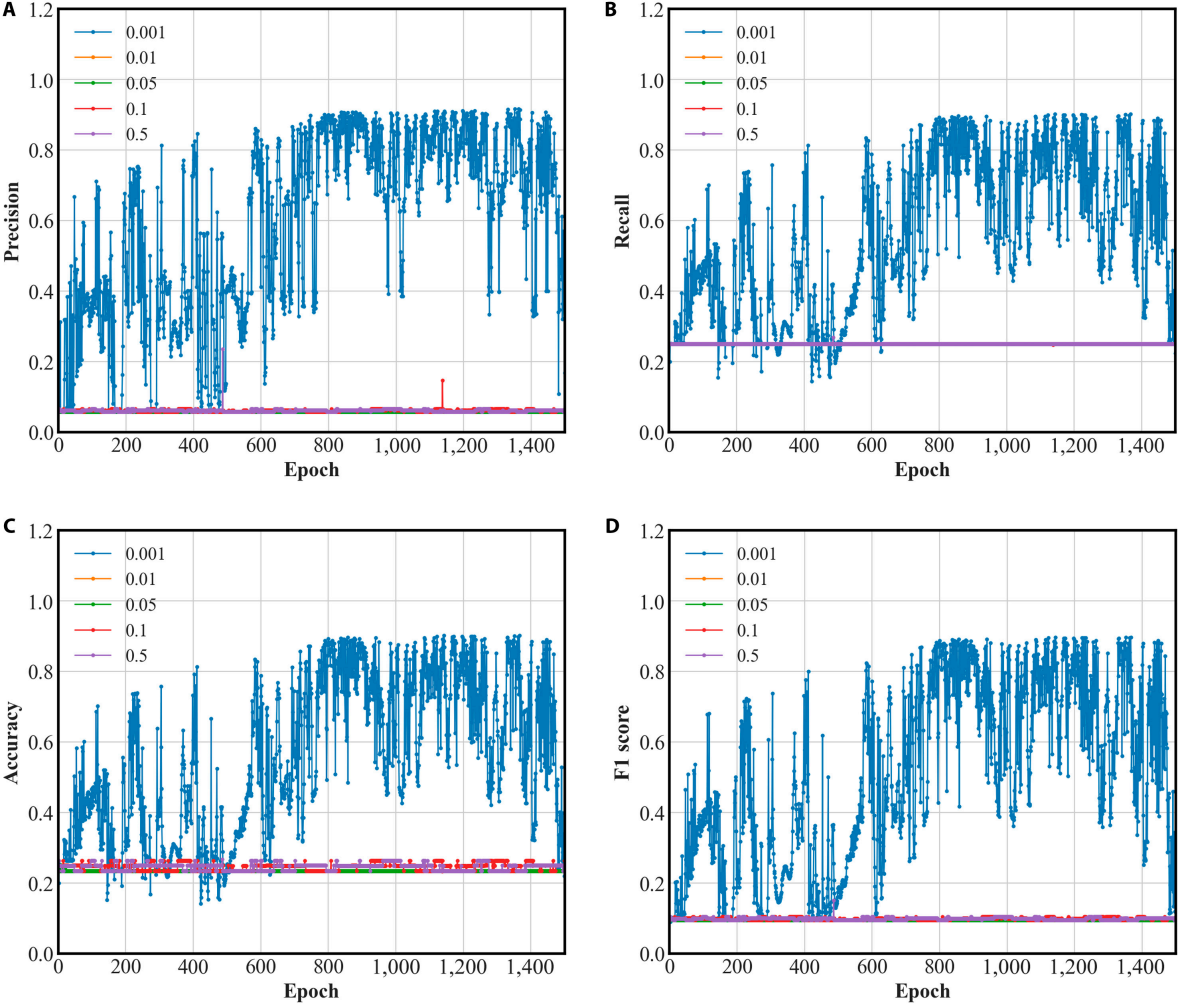
The influence of learning rate on the DGMMF model. (A) Precision, (B) recall, (C) accuracy, and (D) F1 score.

Figure 7 shows the performance of the DGMMF model at different learning rates. We found that when the learning rate is too large, the DGMMF model fails to train. When the learning rate is 0.001, DGMMF is trained successfully. Therefore, we need to choose an appropriate learning rate according to the complexity of the model.

### Experimental results compared to other methods

We compare the experimental results of the DGMMF model with the following model.

• Att-MBiGRU [34]: Multilayer bidirectional gated recurrent units with attention mechanism (Att-MBiGRU) combined convolutional neural network, recurrent neural network, and attention mechanism to realize automatic diagnosis of bearing signals.
• SAE [38]: The stacked autoencoder (SAE) diagnose bearing faults through multiple encoding and decoding operations.
• NN [39]: The neural network (NN) uses neural units and activation functions to diagnose bearing faults.

**Fig. 8.**
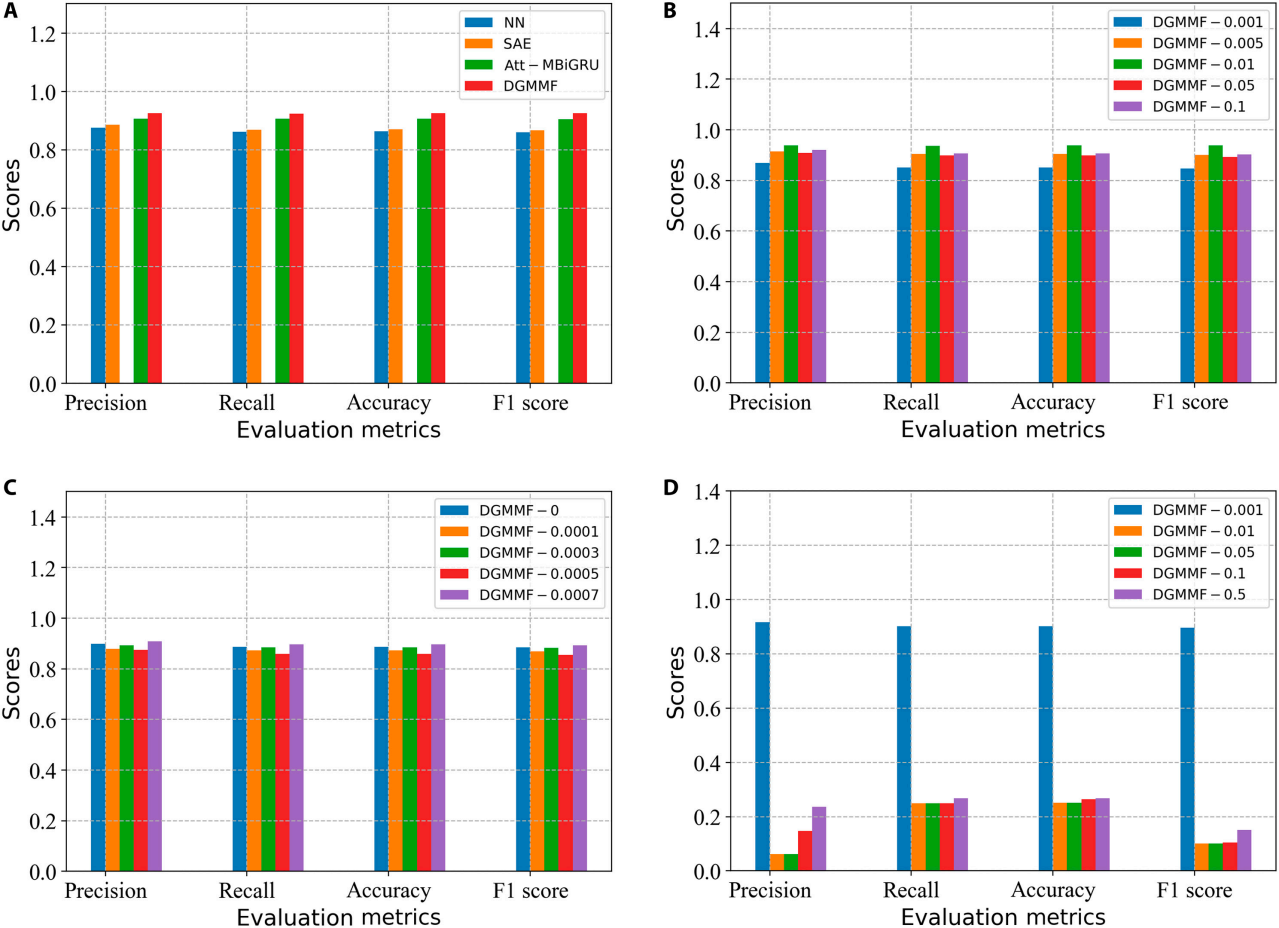
The experimental results of the DGMMF model. (A) The experimental results of the DGMMF model and benchmarks. (B) The experimental results of the DGMMF model with different *α* (maximum value in 1,500 iterations). (C) The experimental results of the DGMMF model with different weight decay (maximum value in 1,500 iterations). (D) The experimental results of the DGMMF model with different learning rate (maximum value in 1,500 iterations).

Table and Fig. 8 show the final experimental results of the above comparison models. Observing the above results, we can find that the DGMMF model has achieved the highest value under all metrics, among which the value of precision is 0.926, the value of recall is 0.924, the value of accuracy is 0.926, and the value of F1 score is 0.925. The second model is the Att-MBiGRU model, and its scores under the 4 evaluation metrics are 0.907, 0.906, 0.907, and 0.905, respectively. The last model is the NN model, and its scores under the 4 evaluation metrics are 0.876, 0.862, 0.863, and 0.860, respectively. Compared with the Att-MBiGRU model, the DGMMF model has an improvement of 2%, 1.9%, 2%, and 2.2% under the 4 evaluation metrics. Compared with the NN model, the DGMMF model has an improvement of 5.7%, 7.1%, 7.3%, and 7.5% under the 4 evaluation metrics.

**Table.** The experimental results of the DGMMF model and benchmarks.

Methods	Precision	Recall	Accuracy	F1 score
NN	0.876	0.862	0.863	0.860
SAE	0.885	0.869	0.870	0.867
Att-MBiGRU	0.907	0.906	0.907	0.905
DGMMF	**0.926**	**0.924**	**0.926**	**0.925**

The above experimental results illustrate the effectiveness of the DGMMF model. The reason why the DGMMF model can achieve better diagnosis results is that the use of VAE as a generative model realizes the augmentation of normal data and fault data, which makes the distribution of various samples balanced, thus simplifying the training difficulty of the model. In addition, the DGMMF model integrates features of different scales. Compared with single-scale features, these multiscale features contain richer information and can better express features, thereby improving the fault diagnosis performance of the model.

## Conclusion

In this work, we design a bearing fault data collection platform based on the IIoT, which is used to collect bearing status data from sensors in real time and feed it back to the bearing fault diagnosis model. We propose a bearing fault diagnosis model DGMMF based on deep generative models with multiscale features. The model can integrate features of different scales and realize the augmentation of normal data and fault data, so that the distribution of various samples is balanced. Finally, we conducted a large number of related experiments on the real bearing fault datasets, which verified the effectiveness of the DGMMF model using multiple evaluation metrics. However, the DGMMF model has not yet been run in a real factory environment. Therefore, in future work, we will look for cooperation opportunities to verify the effectiveness of the DGMMF model in a real factory environment.

## Acknowledgments

**Author Contributions:** Hexuan Hu constructed the model framework, completed some experiments, and double-checked this paper carefully before submission. Yicheng Cai performed the research, analyzed the data, and wrote this paper. Qiang Hu contributed to the drawing illustrations and polishing the language. Ye Zhang contributed to refining the ideas, carrying out additional analyses and finalizing this paper. **Competing interests:** The authors declare that they have no known competing financial interests or personal relationships that could have appeared to influence the work reported in this paper.

## Data Availability

The data used to support the findings of this study are available from the corresponding author upon request.
